# Rapid detection of laboratory cross-contamination with *Mycobacterium tuberculosis *using multispacer sequence typing

**DOI:** 10.1186/1471-2180-9-47

**Published:** 2009-03-03

**Authors:** Zoheira Djelouadji, Jean Orehek, Michel Drancourt

**Affiliations:** 1 Unité de Recherche sur les Maladies Infectieuses et Tropicales Emergentes, UMR CNRS 6236, IRD 3R198, Université de la Méditerranée, IFR 48, Faculté de Médecine, Marseille, France; 2 Département des Maladies Respiratoires, Hôpital Sainte-Marguerite, Assistance Publique-Hopitaux de Marseille, Marseille, France

## Abstract

**Background:**

The ability to culture *Mycobacterium tuberculosis *from clinical specimens serves as the gold standard for the diagnosis of tuberculosis. However, a number of false-positive diagnoses may be due to cross-contamination of such specimens. We herein investigate such episode of cross-contamination by using a technique known as multispacer sequence typing (MST). This technique was applied to six *M. tuberculosis *isolates prepared within the same laboratory over a two-week period of time.

**Results:**

MST analysis indicated a unique and common sequence profile between a strain isolated from a patient with proven pulmonary tuberculosis and a strain isolated from a patient diagnosed with lung carcinoma. Using this approach, we were able to provide a clear demonstration of laboratory cross-contamination within just four working days. Further epidemiological investigations revealed that the two isolates were processed for culture on the same day.

**Conclusion:**

The application of MST has been demonstrated to serve as a rapid and efficient method to investigate cases of possible cross-contamination with *M. tuberculosis*.

## Background

The isolation of *Mycobacterium tuberculosis *complex organisms from clinical specimens collected from suspected patients serves as the gold standard for the proper diagnosis of tuberculosis in the laboratory [1]. However, false-positive cultures have been reported that result from the cross-contamination of specimens via a contaminated bronchoscope [2,3] or, more often, by laboratory cross-contamination [4]. The latter situation has been reported at a frequency ranging from 0.1% to 3% of *M. tuberculosis *[1,4-8]. Laboratory cross-contamination should be suspected when *M. tuberculosis *is cultured from a smear-negative specimen processed in the same batch as a culture from a smear-positive specimen. The factors that increase the likelihood of cross-contamination include instances when only one of several specimens from the same patient is culture-positive and instances when the clinician is considering a diagnosis other than tuberculosis, which the clinician believes to be more likely based on clinical observations [8]. Such false-positives resulting from cross-contaminated specimens are disadvantageous since, besides resulting in a misdiagnosis, they result in unnecessary treatment and delay further diagnostic investigations in an effort to derive a definitive and correct diagnosis [9]. Finally, these false-positive cultures lead to an overestimation of the incidence and prevalence of tuberculosis in humans [10].

A definitive demonstration of cross-contamination can be derived from precise molecular analyses of *M. tuberculosis *isolates. *M. tuberculosis *isolates harbouring identical genotypes are regarded as clones and are thus epidemiologically linked [11]. The most widely used technique for determining the genotype of *M. tuberculosis *is a technique known as IS*6110*-restriction fragment length polymorphism (RFLP) analysis. RFLP analysis requires a large amount of biological material and, thus, poses a risk to laboratory workers due to the harmful nature of this pathogen. Moreover, the latter method requires a substantial amount of time due to the fastidious nature of *M. tuberculosis *[12]. More importantly from, a strictly technical perspective, IS*6110*-RFLP analysis does a poor job of indicating the presence of *M. tuberculosis *when these organisms contain only a few copies of the IS*6110 *sequence [13]. Recently, the variable number tandem repeat (VNTR) PCR-based technique and the mycobacterial interspersed repetitive unit (MIRU) [14] technique have proven to be reliable methods for the resolution of cross-contamination events [15,16].

We herein report the application of a new PCR-sequencing-based genotyping method, known as multispacer sequence typing (MST)[17], for determining whether specimens have been cross-contaminated with *M. tuberculosis *in the laboratory.

### Case report

A 60-year-old man was admitted for an examination to determine whether he had interstitial pneumonia. The patient had been previously hospitalised for two weeks at a different location with symptoms that included shortness of breath, a fever of 38.5°C, and a 7 kg loss of weight within the past month. At the aforementioned hospital, a chest radiograph indicated the presence of bilateral interstitial pneumonia. Subsequent microbiological investigations, including Ziehl-Neelsen staining and a PCR-based assay to test for the presence of *M. tuberculosis *on expectoration, indicated that there were no signs of such an infection. The patient was then transferred to our department for further evaluation. Clinical examination of the patient verified both a body temperature of 38 – 38.5°C and dyspnoea with 90% oxygen saturation under 6 L/min oxygen. The medical history of the patient was unremarkable, except for previous treatment for arterial hypertension. The total body tomodensitometry indicated the presence of nodules in both lungs, in the mediastinal lymph nodes, and in a right axilar lymph node. The pertinent laboratory assays were performed and indicated a value of 5.9 leucocytes/ml with 76% polymorphonuclear cells and 190 platelets/ml. The erythrocyte sedimentation rate was determined to be 28 mm for the first hour. The lactate dehydrogenase level was 612 IU/ml (normal levels are < 430 IU/ml), the gamma GT level was 699 IU/ml (normal levels are < 55 IU/ml), the bilirubin concentration was 13 μmol/l, the AST level was 96 IU/l (normal values are < 25 IU/ml), and the ALT level was shown to be 127 IU/l (normal values are < 45 IU/ml). It was suspected that the patient had already begun to develop pulmonary tuberculosis and thus was recommended to receive anti-tuberculosis therapy since it was reported that *M. tuberculosis *was isolated from an expectoration that was collected 14 days prior during the first hospital visit. Due to the observation that the patient's respiratory status had worsened, the patient was admitted into an intensive care unit for a period of four days. The results of direct microscopic examinations using Gram and Ziehl-Neelsen staining of a surgical lung biopsy were negative. This sample, cultured in BACTEC (Becton and Dickinson, Le Pont de La Claix, France) and in 5% blood agar in slant tubes (Labo Moderne, Dinan, France), remained sterile after a two-month incubation period. Subsequent histological examination discovered large B-cell lymphoma and further assessments confirmed that the patient had stage IV lymphoma that involved the lung, liver, and bone marrow. The patient then received the appropriate anti-lymphoma therapy.

## Results and Discussion

**Figure 1:**
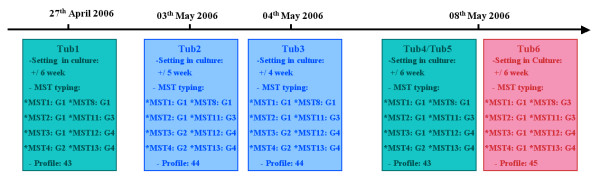
**Distribution of the MST profiles among *M. tuberculosis *isolates performed at different times in a laboratory**.

Our investigation revealed isolation of a total of six *M. tuberculosis *strains from a laboratory that performed analyses for six different patients (including the index patient) within a 2-week period before and after the isolation of *M. tuberculosis *from the index patient (Figure 1). All isolates were recovered from respiratory tract specimens and identified as *M. tuberculosis *by phenotypic methods and the ETR-D sequencing method [18]. Isolate Tub1 (patient A) was recovered from a specimen received and handled on April 27^th^, while isolate Tub2 (patient B) was recovered from a specimen received on May 3^rd^, but handled for setting in culture on May 4^th^. Isolate Tub3 (index patient C) was recovered from a specimen received and handled on May 4^th^, while isolates Tub4, Tub5, and Tub6 (patients D, E, and F, respectively) were recovered from specimens received and handled on May 8^th^. Ziehl-Neelsen staining was performed on all six specimens and the subsequent analyses revealed the presence of acid-fast bacilli for all samples with the exception of the specimen collected from index patient C, which exhibited no acid-fast bacillus. Epidemiological investigation indicated that patients A, D, and E resided in the same ward, whereas no epidemiological link was found between the other three patients, including index patient C.

Eight intergenic spacers were PCR amplified for each of the six *M. tuberculosis *isolates and yielded PCR products of the expected sizes. Sequences derived from these PCR products were combined and assembled for each of the six isolates. MST analysis was completed within four working days. Analysis of the sequence combinations determined three new genetic profiles, including profile ST43, which characterized the three isolates derived from patients A, D, and E; profile ST44, which characterized the two isolates derived from patient B and the index patient C; and profile ST45, which was discovered in the isolate derived from patient F (Figure 1). These new profiles resulted from a novel combination of the following spacer alleles: the ST43 profile combined alleles 1/MST1, 1/MST2, 1/MST3, 2/MST4, 1/MST8, 3/MST11, 4/MST12, and allele 4/MST13; the ST44 profile combined alleles 1/MST1, 1/MST2, 2/MST3, 2/MST4, 1/MST8, 3/MST11, 4/MST12, and allele 4/MST13; and the ST45 profile combined alleles 1/MST1, 1/MST2, 1/MST3, 1/MST4, 3/MST8, 3/MST11, 4/MST12, and allele 4/MST13. The profiles for ST43, ST44, and ST45 have been added to our free and accessible MST database http://ifr48.timone.univ-mrs.fr/MST_Mtuberculosis/mst. MST genotyping data were assumed to be authentic based on the observations that the PCR-negative controls remained negative, coupled with the observation that all PCR products were of the predicted size. Moreover, analysis of the spacer sequences edited in this work identified three new profiles, clearly indicating that amplicons did not result from laboratory contamination as a consequence of previous experiments.

The MST genotyping data provided evidence to support epidemiological and clinical data that confirmed laboratory cross-contamination. Specifically, one profile (ST43) comprised three isolates recovered from epidemiologically-linked patients, whereas a different profile (ST45) characterized only one isolate from a specimen collected from an unrelated patient F. The profile ST44 was discovered for two *M. tuberculosis *isolates obtained from the index patient C and one unrelated patient B. Microscopic examination of a respiratory tract specimen collected from patient B indicated the presence of acid-fast bacilli, while the same analysis performed for a specimen from the respiratory tract of the index patient C showed no indication of acid-fast bacilli. Both of the latter two specimens were handled in the same laboratory, on the same day, and within the same batch of sample preparations, which explains the observation that the specimen recovered from the index patient (patient C) was contaminated by the specimen collected from patient B. Such a situation has been previously observed in cases of laboratory cross-contamination [19,20]. Interestingly, the frequency of false-positive cultures has been shown to be higher for laboratories that do not process high numbers of specimens [6], as was the case in the present report. As an example, in the laboratory setting, cross-contamination events may occur in the safety cabinet when a smear-positive specimen is handled in parallel with a smear-negative specimen, or during the phenotypic identification of isolates during the niacin test [15]. Cross-contamination of respiratory tract specimens by the avirulent *M. tuberculosis *H37Ra reference strain has also been reported [21].

The MST method, which was used in this study in addition to the more commonly used VNTR/MIRU typing method [15,16], requires a relatively small amount of sample DNA from the patient. In contrast to the conventional IS*6110*-RFLP method, which requires a relatively large amount of DNA, both the MST and the VNTR/MIRU typing methods require only small DNA samples as they are based on PCR amplification of selected genomic regions [22]. The fact that such a small amount of material is handled during these aforementioned procedures is an obvious advantage, since it limits the risk of exposure of laboratory personnel to a dangerous pathogen. Since the MST method is based on sequence analysis, is reproducible and is easily exchangeable, we propose and offer a free and accessible *M. tuberculosis *MST database (at http://ifr48.timone.univ-mrs.fr/MST_MTuberculosis/mst) so that microbiologists may compare the spacer sequence profiles they obtain with previously determined profiles for *M. tuberculosis*. The requirement for sequence analysis may limit the diffusion of MST to those laboratories that are equipped with an automatic sequencer, which is not a commonality in most laboratories, especially those in resource-limited countries.

Since MST uses PCR amplification as the first experimental step, it has the advantage of being applicable to DNA extracts from inactivated mycobacterial cultures [23] shortly after they are shown to be positive. The MST results were obtained in four working days (from the moment the culture was obtained to the interpretation of MST analysis). A similar, yet slightly longer delay of 13 days (median value) between initial analysis and interpretation of results was recently reported when using the VNTR/MIRU method. In contrast, the conventional IS*6110 *technique provided results in a median time of 45 days [16]. The delay period required to complete the MST analysis is certainly short enough to contribute to the interpretation of laboratory data that may have a significant clinical impact on patients.

## Conclusion

Our report confirms the importance of rapid identification of cross-contamination. Indeed, the misdiagnosed patient received unnecessary anti-tuberculosis therapy and the final correct diagnosis was slightly delayed. MST typing proved to be an efficient new tool for the detection of cross-contamination with *M. tuberculosis*. In addition, MST results may be obtained within a few days, which significantly improves the quality of laboratory processing and, therefore, the quality of medical care for the patient.

## Methods

### Epidemiological investigation

We reviewed laboratory charts to identify mycobacterial isolates that were identified as *M. tuberculosis *during the 2-week period before and after the isolation of *M. tuberculosis *in the index patient (total study period, 4 weeks). We carefully reviewed the batch number of each of these isolates in order to pinpoint the day on which clinical specimens were handled for setting in culture, as well as any epidemiological links between patients.

### Multispacer sequence typing

Isolates were identified using conventional methods [24] and, after proper inactivation [23], by sequencing of the ETR-D spacer, as previously described [18]. The MST genotyping, PCR amplification and sequence analysis of eight intergenic spacers were performed as described previously [17]. Two negative controls consisting of the PCR mix in the absence of the target DNA template were also performed. Purified PCR products were sequenced by use of the BigDye Terminator 1.1 Cycle Sequencing kit (Applied Biosystems, Courtaboeuf, France). Sequencing electrophoresis was performed using a 3130 Genetic Analyser (Applied Biosystems). Sequences were aligned using CLUSTAL W http://pbil.ibcp.fr and compared to each other and with a local database of *M. tuberculosis *spacer sequences that is freely available on our website http://ifr48.timone.univ-mrs.fr/MST_Mtuberculosis/mst. This study was approved by the local Ethics Committee, Marseille, France.

## Competing interests

The authors declare that they are the inventors of a protective patent on this matter deposited by the Mediterranée University, Marseilles, France.

## Authors' contributions

DZ performed the described experiments, analysed the results and wrote the manuscript. JO performed the epidemiological investigation. MD analysed the results and contributed to drafting of the manuscript.
